# Impact of general anesthesia on ablation catheter stability during pulmonary vein isolation based on a novel measurement approach

**DOI:** 10.1038/s41598-023-44450-7

**Published:** 2023-10-11

**Authors:** Shimpei Kuno, Yusuke Nakano, Yasushi Suzuki, Hirohiko Ando, Wataru Suzuki, Hiroshi Takahashi, Tetsuya Amano

**Affiliations:** 1 Department of Cardiology, Aichi Medical University, 1-1 Yazakokarimata, Nagakute, Aichi 480-1195 Japan; 2 Fujita Health University School of Medical Science, 1-98 Dengakukubo, Kutsukake, Toyoake, Aichi Japan

**Keywords:** Cardiology, Medical research

## Abstract

Catheter ablation for atrial fibrillation (AF) during pulmonary vein isolation (PVI) is performed under general anesthesia (GA) or conscious sedation (CS). GA during PVI may improve treatment outcomes by improving catheter stability. However, the magnitude of GA-derived catheter stability compared with that of CS is unclear. We directly assessed catheter movement and determined the impact of GA compared with that of CS on ablation catheter stability during PVI. Patients who underwent initial ablation using the EnSite Precision™ mapping system were recruited and divided into two groups (GA and CS groups). The two groups were compared for ablation catheter stability during PVI based on the distance traveled by the catheter distal tip per second, clinical periprocedural characteristics, and periprocedural complications. Among 69 consecutively admitted patients, data of 30 patients (17 in the GA group and 13 in the CS group) and the distance traveled per second by the catheter on 148,976 points/patient were evaluated. The GA group had a significantly smaller catheter tip travel distance than the CS group (0.92 [0.82‒1.16] vs. 1.25 [1.14‒1.38], *p* = 0.01). Therefore, GA during PVI for AF provides greater catheter stability than CS and will contribute to more accessible and safer PVI procedures.

## Introduction

Atrial fibrillation (AF) is the most common tachyarrhythmia in daily clinical practice and is associated with an increased risk of heart failure, stroke, and mortality^1^. Haissaguerre et al*.* first demonstrated that most atrial premature beats initiate frequent paroxysms of AF in the pulmonary veins (PVs)^2^^,^^3^. Therefore, radiofrequency ablation for AF is now achieved by encircling ipsilateral PVs to electrically isolate the PVs, termed pulmonary vein isolation (PVI)^4^^,^^5^. The indications for catheter ablation in AF cases have expanded, as evidence for the efficacy and safety of PVI for AF has been validated. AF ablation is currently the most common electrophysiological treatment worldwide^6^.

**Figure 1:**
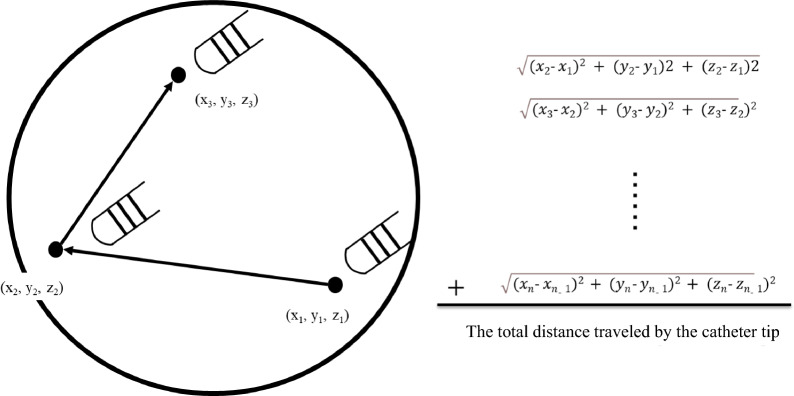
Evaluation of the distance traveled by the catheter tip. The distance traveled by the catheter tip was calculated from the X/Y/Z axis coordinate information recorded at 103 Hz by the EnSite Precision system. The total distance traveled by the catheter tip per second was calculated for each ablation region area by AutoMark^TM^, and the average for each area was calculated.

**Figure 2:**
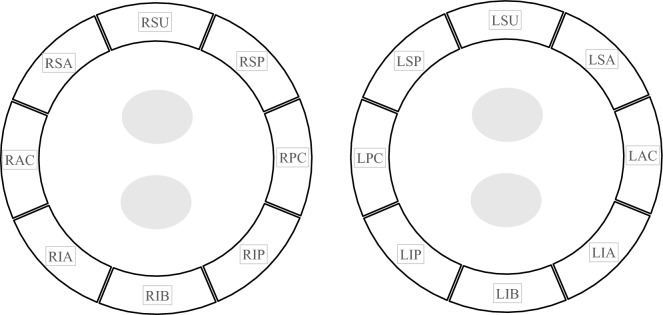
PVI line classified into 16 regions. The gray circle indicates the pulmonary vein ostium. The pulmonary vein isolation line was classified into 16 regions. In each region, the total distance traveled by the catheter tip per second was calculated and compared between the general anesthesia and conscious sedation groups. Left superior anterior (LSA), Left anterior carina (LAC), Left inferior anterior (LIA), Left inferior bottom (LIB), Left inferior posterior (LIP), Left posterior carina (LPC), Left superior posterior (LSP), Left superior upper (LSU), Right superior anterior (RSA), Right anterior carina (RAC), Right inferior anterior (RIA), Right inferior bottom (RIB), Right inferior posterior (RIP), Right posterior carina (RPC), Right superior posterior (RSP), Right superior upper (RSU).

PVI is usually performed under general anesthesia (GA) or conscious sedation (CS). A previous report demonstrated that the use of GA during PVI reduces the prevalence of PV reconnection observed at the time of repeat ablation^7^. Therefore, GA is widely used for PVI of AF across many centers in the United States^8^. Some studies have reported that the contact force (CF) of the ablation catheter to the heart remains highly variable during PVI despite efforts to optimize it; this may be due to respiratory movements^9^. In theory, GA contributes to better stability of the CF of the catheter and lesion formation during PVI via appropriate respiration management. However, there are no reports evaluating the relationship between the method of anesthesia and ablation catheter stability via direct assessment during PVI. In this study, we introduced a new method to directly assess catheter movement and evaluated the influence of GA compared with that of CS on ablation catheter stability during PVI (Figs. 1 and 2).

## Results

### Baseline patient characteristics

**Figure 3:**
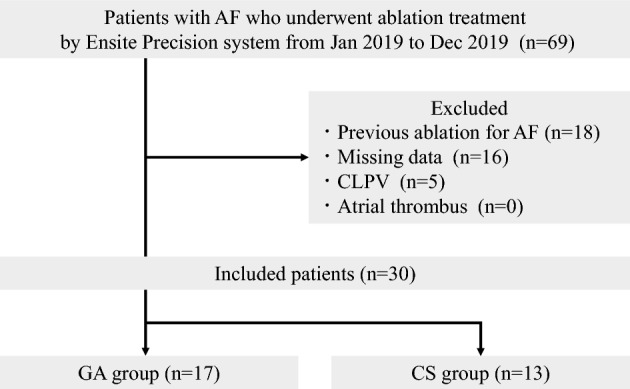
Flow diagram for patient selection. Patients with atrial fibrillation (AF) who underwent ablation treatment using the EnSite Precision system between January 1, 2019 and December 31, 2019 were included in this study. Patients were excluded from this study if they had undergone previous ablation treatment, had missing data, or had a common left pulmonary vein (CLPV). Patients included in this study were then classified into either the General anesthesia (GA) or Conscious sedation (CS) groups according to the method of anesthesia. .

Among 69 consecutively admitted patients, 30 (17 in the GA group and 13 in the CS group) were enrolled in this study. The exclusion criteria included patients with previous ablation for AF, missing data about the X/Y/Z axis coordinate information of the ablation catheter during PVI, a common left pulmonary vein (CLPV), and documented atrial thrombus. A flow diagram for patient selection is shown in Fig. 3. The baseline patient characteristics are summarized in Table 1. The mean age was 67.2 ± 8.5 years in the GA group and 65.9 ± 11.0 years in the CS group (*p* = 0.71). Moreover, 47.1% of the participants in the GA group and 69.2% in the CS group were male individuals (*p* = 0.23). Body mass index (BMI) was 24.3 ± 5.4 kg/m^2^ in the GA group and 22.8 ± 4.3 kg/m^2^ in the CS group (*p* = 0.42). Two patients had sleep apnea syndrome (SAS) in the GA group (11.7% vs. 0.0%; *p* = 0.49). There were no significant differences in other variables, including percentage of paroxysmal AF, and CHA2DS2-VASc score, between the two groups.

**Table 1:** Clinical characteristics and echocardiographic measurements.

Characteristics	General anesthesia (n = 17)	Conscious sedation (n = 13)	*p*
Age, years	67.2 ± 8.5	65.9 ± 11.0	0.71
Male sex, n (%)	8 (47.1%)	9 (69.2%)	0.23
Body mass index, kg/m^2^	24.3 ± 5.4	22.8 ± 4.3	0.42
Paroxysmal atrial fibrillation, n (%)	9 (52.9%)	8 (61.5%)	0.64
CHA2DS2-VASc score	2.71 ± 1.76	1.77 ± 1.30	0.11
Chronic heart failure, n (%)	4 (23.5%)	0	0.06
Chronic kidney disease, n (%)	1 (5.9%)	0	0.37
Hypertension, n (%)	9 (52.9%)	9 (69.2%)	0.37
Diabetes mellitus, n (%)	3 (17.6%)	0	0.11
Prior stroke, n (%)	3 (17.6%)	1 (7.7%)	0.43
Sleep apnea syndrome, n (%)	2 (11.7%)	0	0.49
Left atrial diameter, mm	36.3 ± 7.4	34.7 ± 6.3	0.52
Ejection fraction, %	61.7 ± 8.8	61.9 ± 7.8	0.96

Values are presented as numbers (%) or means ± standard deviations. The CHA2DS2-VASc score is a clinical estimation of the risk of stroke in patients with atrial fibrillation; scores range from 0 to 9, with higher scores indicating a greater risk of stroke. *p* < 0.05 was considered statistically significant. Data were compared using the Student’s t-test. Categorical variables are presented as patient numbers (%) and analyzed using the chi-squared and Fisher’s exact tests to examine differences between the groups.

### Periprocedural characteristics

Procedure-related data are presented in Table 2. PVI was successful in all patients, and a bidirectional block was confirmed. The entry-to-exit time, ablation time, and fluoroscopic time were not significantly different between the two groups. Substrate modification, including cavotricuspid isthmus ablation and superior vena cava isolation, was performed similarly between the two groups. There was no significant difference between the two groups, as added in Table 2, in the percentage of first-pass (Table 2). Regarding vital signs, the variability in the respiratory rate in terms of either the standard deviation (SD) or SD/mean index was significantly smaller in the GA group; however, the other parameters were not significantly different between the two groups.

**Table 2:** Periprocedural characteristics and variability of vital signs.

Characteristics	General anesthesia (n = 17)	Conscious sedation (n = 13)	*p*
Acute success of pulmonary vein isolation, n (%)	17 (100%)	13 (100%)	0.99
Entry-to-exit time, min	186 ± 44	200 ± 46	0.42
Ablation time, min	135 ± 41	154 ± 44	0.22
Fluoroscopic time, min	24.8 ± 16	34 ± 17	0.12
pulmonary vein isolation time, min	58.1 ± 19	80.9 ± 27	0.13
Additional ablation, n (%)	7 (41.1%)	3 (23.1%)	0.44
Cavotricuspid isthmus ablation, n (%)	6 (35.3%)	2 (15.4%)	–
Superior vena cava isolation, n (%)	1 (5.9%)	1 (7.7%)	–
First-pass isolation, n (%)	13 (76.4%)	10 (75.9%)	0.66
Variability of vital signs			
Respiration rate, standard deviation	0.00 (0.00‒0.89)	2.70 (1.67–3.50)	< 0.01
SpO2, standard deviation	0.00 (0.00‒0.44)	0.40 (0.00‒0.72)	0.17
Mean blood pressure, Standard deviation	10.71 (9.07‒13.22)	12.19 (8.79‒14.95)	0.51
Heart rate, standard deviation	11.43 (4.95‒16.06)	8.69 (5.15‒12.99)	0.65
Adjusted variability of vital signs			
Respiration rate, standard deviation/mean	0.00 (0.00‒0.084)	0.20 (0.12‒0.29)	< 0.01
SpO2, standard deviation/mean	0.00 (0.00‒0.00)	0.00 (0.00‒0.01)	0.17
Mean blood pressure, standard deviation/mean	0.16 (0.12‒0.18)	0.12 (0.09‒0.17)	0.1
Heart rate, standard deviation/mean	0.17 (0.08‒0.22)	0.11 (0.07‒0.18)	0.43

Values are presented as numbers (%), means ± standard deviations, or medians (interquartile ranges). *p* < 0.05 was considered statistically significant. Data were compared using the Student’s t-test. Categorical variables are presented as patient numbers (%) and analyzed using the chi-squared and Fisher’s exact tests to examine differences between the groups.

### Distance traveled by the distal tip of the catheter

**Figure 4:**
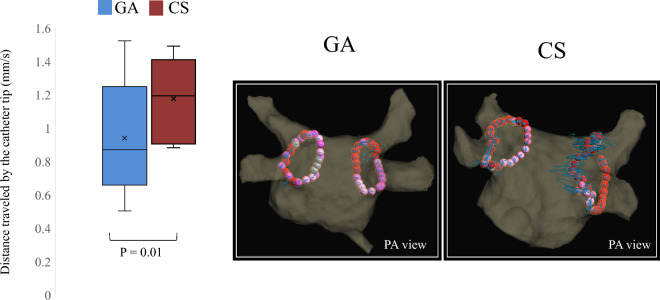
Distance traveled by the catheter during PVI under GA or CS. (**a**) Comparison of the overall PVI session. (**b**) A representative case of an AutoTrack map that was used in this study. (**a**) The three-dimensional coordinate data used to calculate the distance traveled by the catheter averaged 148,976 points for each patient. The distance traveled by the catheter during pulmonary vein isolation (PVI) ablation was significantly smaller in general anesthesia (GA) than in conscious sedation (CS). *p* < 0.05 was considered statistically significant. Boxplots report the 25% (lower hinge), 50% (center line) and 75% (upper hinge) quantiles. Whiskers indicate observations equal to or outside the hinge ± 1.5 × the IQR. The distance traveled by the catheter tip were compared using the Mann–Whitney U test. (**b**) The AutoTracks map shows the movement of the ablation catheter during radio frequency (RF). Ablation lesions were marked in different colors depending on the lesion index (LSI; a multi-parametric index incorporating contact force and radiofrequency current data across time) as follows: white (> 3.0, < 4.0); pink (> 4.0, < 4.8); red (> 4.8) (If the esophageal temperature increased and the ablation was stopped before the LSI reached 3.0, a gray automatic mark was applied manually.) The blue line shows the trajectory of the ablation catheter tip. Continuity and ablation catheter stability can be evaluated for each region. Posterior-anterior view (PA view).

**Figure 5:**
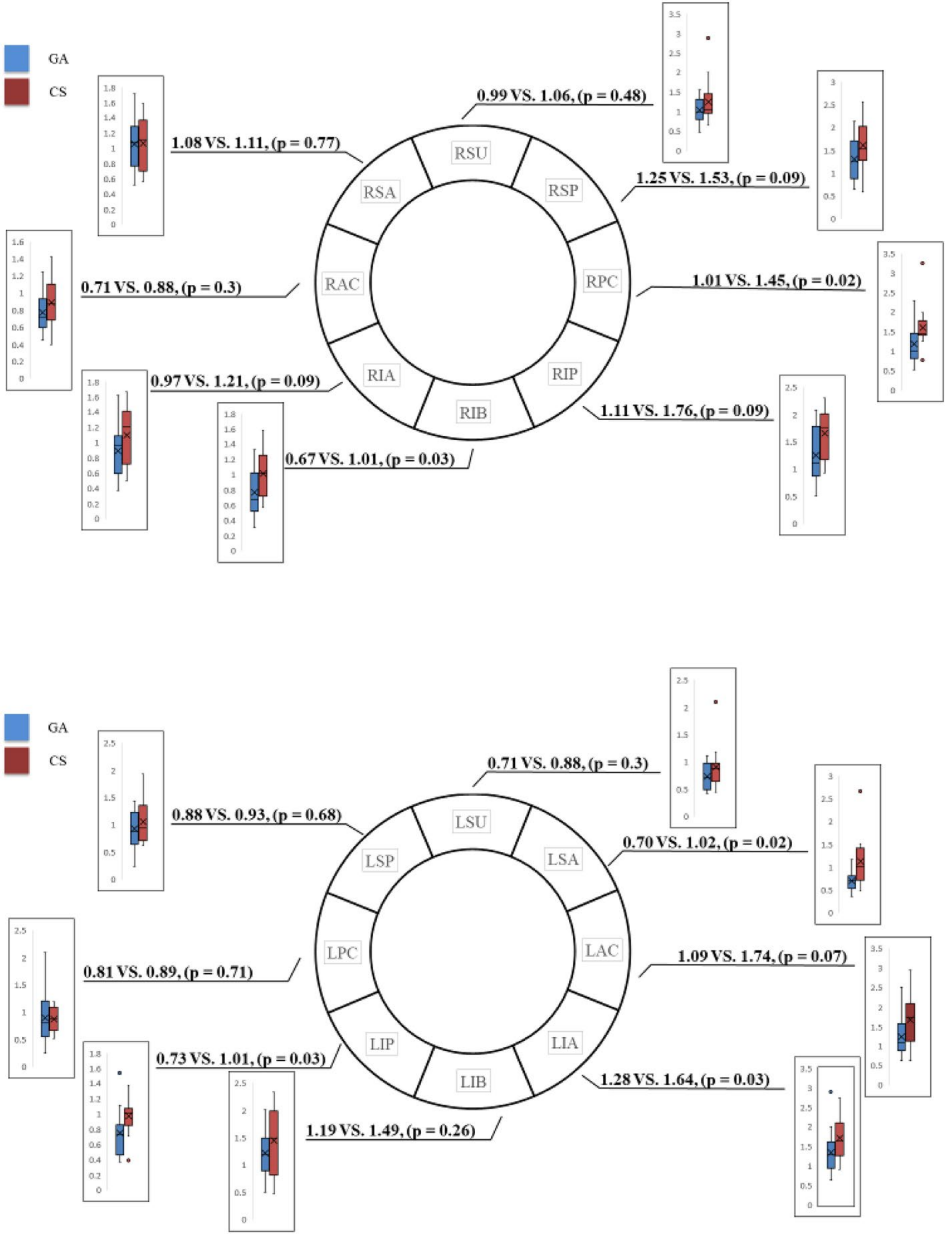
Comparison by each region with respect to the distance traveled by the catheter for PVI ablation performed under GA or CS. Comparison of each region based on the distance traveled by the catheter for pulmonary vein isolation (PVI) ablation performed under general anesthesia (GA) or conscious sedation (CS). The vertical axis of the box-and-whisker plot represents the distance traveled by the catheter (mm/s). In all regions, especially in the left inferior anterior (LIA), left inferior posterior (LIP), left superior anterior (LSA), right inferior bottom (RIB), and right posterior carina (RPC), the distance traveled by the catheter tip was smaller in the GA group than in the CS group. Values are presented as medians (interquartile ranges). *p* < 0.05 was considered statistically significant. Boxplots report the 25% (lower hinge), 50% (center line) and 75% (upper hinge) quantiles. Whiskers indicate observations equal to or outside the hinge ± 1.5 × the IQR. The distance traveled by the catheter tip were compared using the Mann–Whitney U test. Left anterior carina (LAC), Left inferior bottom (LIB), Left posterior carina (LPC), Left superior posterior (LSP), Left superior upper (LSU), Pulmonary vein isolation (PVI), Right superior anterior (RSA), Right anterior carina (RAC), Right inferior anterior (RIA), Right inferior posterior (RIP), Right superior posterior (RSP), Right superior upper (RSU).

The three-dimensional coordinate data used to calculate the distance traveled by the catheter averaged 148,976 points for each patient. A comparison of the distance traveled by the catheter distal tip per second, indicating ablation catheter stability during PVI, between the GA and CS groups is shown in Fig. 4a. Overall, the distance traveled by the catheter during PVI ablation was significantly shorter in the GA group than in the CS group (0.92 [0.82‒1.16] vs. 1.25 [1.14‒1.38], *p* = 0.01). Figure 4b is a representative image of the Auto Track map used to compare the distance traveled by the catheter between the two groups. A detailed analysis of the data for each region is shown in Fig. 5. In all regions, especially in the left inferior anterior (LIA), left inferior posterior (LIP), left superior anterior (LSA), right inferior bottom (RIB), and right posterior carina (RPC), the distance traveled by the catheter tip was shorter in the GA group than in the CS group (1.28 [0.95‒1.62] vs. 1.64 [1.27‒2.10], *p* = 0.03; 0.73 [0.46‒0.86] vs. 1.01 [0.85‒1.07], *p* = 0.03; 0.70 [0.53‒0.81] vs. 1.02 [0.71‒1.42], *p* = 0.02; 0.67 [0.52‒1.02] vs. 1.01 [0.72‒1.25], *p* = 0.03; and 1.01 [0.81‒1.46] vs. 1.45 [1.41‒1.77], *p* = 0.02, respectively).

### Periprocedural complications

No serious periprocedural complications, such as stroke, cardiac tamponade, symptomatic severe PV stenosis, atrio-esophageal fistula, or re-intubation, were observed in both groups.

## Discussion

To the best of our knowledge, this is the first study to compare directly assessed catheter stability between GA and CS. In this study, we quantitatively evaluated the impact of GA compared with that of CS on ablation catheter stability during PVI using 148,976 points/patient data obtained from 30 patients. Our major findings are as follows: (1) Ablation catheter stability during PVI was significantly superior in the GA group than in the CS group, and (2) the distance traveled by the ablation catheter was shorter in all regions around PV in the GA group. In particular, in the GA group, the LIA, LIP, LSA, RIB, and RPC had significantly lower catheter variability. Previous studies have demonstrated the effectiveness of GA for PVI. However, the reasons for this have not yet been fully elucidated. DiBiase et al*.* demonstrated that PVI under GA was associated with better treatment outcomes with a single procedure, and GA reduced the prevalence of PV reconnection at the time of repeat ablation^7^. They hypothesized that GA helps improve catheter stability and optimizes radiofrequency energy delivery. CF-guided PVI achieved a high success rate for durably ablated lesions^10^. Chikata et al*.* demonstrated that GA was associated with lower AF recurrence in patients with paroxysmal AF (*p* = 0.022) and that CF parameters (CF, force–time integral [FTI], and FTI/wall thickness) improved in the GA group^11^. However, CF is an indirect indicator of durable lesions. This is because the target CF value differs among patients, and the magnitude of the CF value itself cannot be used to evaluate catheter stability, i.e., the degree of catheter movement.

In this study, we evaluated catheter stability using three-dimensional coordinate data as a new method and showed that GA improved catheter stability in PVI. The reasons for this may include the following: First, anatomic factors may have contributed to poor catheter stability. Muscular folds between the left PVs and left atrial appendage can often be narrow with an acute angle, making it difficult to stabilize a catheter onto the LSA during catheter ablation^12^. The LSA is known to be one of the areas where PV reconnection is most likely to occur^13^. Importantly, in this study, we found a significant difference in the catheter stability in this area, as determined by our new method. Second, the pain due to ablation may have resulted in poor stability. Attanansio et al*.* compared pain reactions during PVI under deep sedation via cryoballoon ablation and radiofrequency ablation (RFA) using pain scales (including the Face, Legs, Activity, Cry, Consolability Behavioral Pain Assessment Scale). They reported that 92% of patients treated with RFA reported having one or more pain reactions during the procedure^14^. Aryana et al*.* recorded episodes of pain during ablation. The overall incidence of pain during RFA delivery to the anterior and posterior walls was 1.0% and 13.1%, respectively^15^. In other words, the posterior wall is a more painful area than the anterior wall. PVI with RFA is often painful; however, pain can be appropriately controlled by GA. It is suspected that catheter stability decreases if the body movements of the patient are accompanied by pain. Finally, a stable respiratory status may have improved catheter stability. Sarkozy et al*.* characterized CF variability and the reasons for variability during PVI in 20 consecutive patients. The main reasons for CF variability are systolo-diastolic heart movement (29%) and respiration (27%)^9^. In our study, among the vital signs (including heart rate, blood pressure, respiratory rate, and SpO_2_) during PVI, only the respiratory rate varied less significantly in the GA group than in the CS group. In other words, appropriate management of respiration by GA may stabilize the catheter. In addition, ablation times in this study tended to be shorter with GA. This supports our hypothesis that stabilization of the catheter in GA facilitates PVI.

The clinical benefits of GA on PVI include improved catheter stability during the procedure via both reduced pain and appropriate management of the respiratory tract, leading to a high success rate for PVI. We believe that this provides a robust rationale for the use of GA in PVI.

This study has some limitations. First, the small sample size could potentially lead to result instability, although we conducted a thorough analysis using a very large amount of three-dimensional coordinate data for each individual case. Second, this was retrospective, single-center design, without randomization, which may introduce the potential variations in patient backgrounds. However, we observed no significant differences in patient backgrounds between the two groups. Nonetheless, it is important to acknowledge that unknown factors could potentially influence the results, and their impact can’t be completely ruled out. Third, when assessing catheter stability and ablation efficacy, it might be preferable to exclude the measurement of travel distances within 5 mm of the target point. However, it was technically unfeasible to exclude data points within 5 mm range the target point in this study. Finally, this is an unprecedented method of analyzing catheter stability. Therefore, there is no established evidence supporting this approach. However, our study analyzed a very large amount of three-dimensional coordinate data that is consistent with the results from previous clinical studies and clinical practice. This may be methodologically correct but requires further verification.

In conclusion, in this study, we demonstrated the superiority of GA over CS for catheter stability using a new evaluation method. This supports the validity of using GA to lead successful PVI.

## Methods

This retrospective observational study included consecutively admitted patients who underwent initial ablation for AF using the EnSite Precision™ mapping system (Abbott, St. Paul, MN) at the Aichi Medical University Hospital between January 1, 2019 and December 31, 2019. Patients were excluded if they had previously undergone ablation treatment, insufficient three-dimensional coordinate data, or a common left PV. The patients were divided into two groups (GA and CS groups), and the ablation catheter stability during PVI was compared based on the distance traveled per second by the catheter distal tip. Anesthesia was administered under GA or CS at the preference of the patient and at the discretion of the attending physician. In addition, the clinical characteristics obtained from reviews of medical charts, periprocedural characteristics, and complications were evaluated.

### Ethics statement

This study was conducted in accordance with the principles of the Declaration of Helsinki. The study protocol was approved by the Ethics Committee of the Aichi Medical University Hospital, and written informed consent was obtained from all patients.

### Pre-procedural evaluation

Antiarrhythmic medication for AF was administered at the discretion of the physician. Oral anticoagulants were administered at least 3 weeks before the procedure, until the day before the procedure, and resumed on the night of the day after the procedure. All patients underwent transesophageal echocardiography (TEE) and computed tomography coronary angiography (CTCA). Catheter ablation was canceled in cases where intra-atrial thrombus was documented by TEE or CTCA.

### General anesthesia

All GA management procedures were performed by an anesthesiologist. At the beginning of the procedure, before femoral vein cannulation, we intravenously administered propofol (Diprivan; 1‒2 mg/kg), remifentanil (Ultiva Intravenous; 0.05‒0.2 μg/kg/min), and rocuronium (Esmeron; 0.6‒0.9 mg/kg) with subsequent orotracheal intubation and mechanical ventilation. Anesthesia was maintained with desflurane (4‒5%) and remifentanil (0.05‒0.2 μg/kg/min), with fentanyl administered as needed. Intraoperative surveillance included general monitoring (electrocardiography, blood pressure, and oxygen saturation by pulse oximeter [SpO_2_], end tidal CO_2_ [EtCO_2_], and bi-spectral index [BIS; a commercial device used to assess the depth of anesthesia] for sedation monitoring and training for monitoring muscle relaxation). The ventilator was set in volume-controlled ventilation mode, with a tidal volume of 6‒8 ml/kg and a ventilation rate determined by reference to EtCO_2_ and partial pressure of CO_2_ values. After the ablation procedure, desflurane and remifentanil were discontinued, and rocuronium was antagonized by sugammadex (Bridion; intravenous 4 mg/kg). Extubation was performed when the assessment of consciousness, spontaneous breathing, and circulation was adequate.

### Conscious sedation

Dexmedetomidine was administered intravenously before the intravenous catheter was inserted. Intraoperative monitoring included that of general parameters (blood pressure, SpO_2_, and EtCO_2_) using electrocardiography. The level of sedation was assessed using the Richmond Agitation-Sedation Scale (RASS) (1 = anxious and agitated, restless; 2 = cooperative, oriented, tranquil; 3 = responsive to verbal commands, drowsy; 4 = asleep, responsive to light stimulation; 5 = asleep, slow response to stimulation; and 6 = no response to stimulation). Dexmedetomidine was administered at an initial rapid dose (1‒5 μg/kg/h) for 30 min, followed by a continuous dose (0.15‒0.3 μg/kg/h). Drug doses were titrated to achieve the targeted sedation level (RASS -2‒-3) by increasing the infusion rate. Fentanyl (50‒100 mg) was administered before ablation started, followed by continuous administration (1‒5 µg/kg/h). The dose of fentanyl was adjusted according to changes in the vital signs of the patient, RASS, and EtCO_2_ monitoring.

### Ablation procedure

A single trans-septal puncture was performed using a radio frequency (RF) needle (NRG™ RF Trans-septal Needle C0; Baylis Medical Company) and SL0 (SL0; Abbott). Subsequently, an 8-Fr long sheath (SL0; Abbott) and Steerable sheath (Agilis™ NxT Steerable Introducer, St. Jude Medical) were placed in the left atrium. Ablation was then started from the right PV using an ablation catheter, guided by three-dimensional-Merge-CT images using the EnSite system. The ablation temperature was limited to 43 °C, and the ablation power was limited to 25 W in the posterior segments and 30 W in the other segments. A luminal esophageal temperature (SensiTherm Multi™; Abbott) of > 40 °C was used to stop the ablation. Circumferential isolation was first performed anatomically using a circular catheter (Advisor VL™; Abbott). The procedure was accomplished if a bidirectional conduction block between the intra-PV and atrium was confirmed. No provocation test for dormant conduction by adenosine triphosphate was performed. First‐pass PVI was defined as the achievement of a successful PVI before initial circle completion.

### Location information of ablation catheter

We describe here a new method of evaluating stability, different from CF and other indirect methods. The X/Y/Z axis coordinate information of the distal electrode of the ablation catheter during PVI was recorded at 103 Hz using the EnSite system. Based on the coordinate information, the distance traveled per second for each ablation region was acquired (Fig. 1). Regions created by ablation were classified into 16 regions (left anterior carina, LIA, left inferior bottom, LIP, left posterior carina, LSA, left superior posterior [LSP], left superior upper, right superior anterior, right anterior carina, right inferior anterior, RIB, right inferior posterior, RPC, right superior posterior, right superior upper), and the average distance traveled per second was obtained for each region (Fig. 2). Only AutoMark™ (an automated ablation lesion tagging on the EnSite Precision system [Abbott]) on the circumference of PVI was employed in the analysis, and AutoMark™ outside the PVI line that could not be classified into the 16 regions mentioned above was excluded from the analysis. The classification of regions and confirmation of the exclusion of regions that were not on the circumference were performed by one physician and two clinical engineers who were blinded to the clinical characteristics of patients.

### Statistical analyses

Continuous variables with a normal distribution are presented as means ± standard deviations. Data were compared using the Student’s t-test. Variables with a non-normal distribution are expressed as medians with interquartile ranges and compared using the Mann–Whitney U test. Categorical variables are presented as patient numbers (%) and analyzed using the chi-squared and Fisher’s exact tests to examine differences between the groups. All *p*-values were two-tailed, with *p* < 0.05 considered statistically significant. Statistical analyses were performed using the SPSS (IBM Corp., Armonk, NY, USA) software.

## Data Availability

The datasets used and/or analyzed during the current study are available from the corresponding author on reasonable request.
